# A narrative review of the CEPH-accredited bachelor’s public health programs’ curricula in the United States

**DOI:** 10.3389/fpubh.2024.1436386

**Published:** 2024-08-21

**Authors:** Satish K. Kedia, Coree Entwistle, Laura Magaña, Tracie G. Seward, Ashish Joshi

**Affiliations:** ^1^School of Public Health, University of Memphis, Memphis, TN, United States; ^2^Association of Schools and Programs of Public Health (ASPPH), Washington, DC, United States

**Keywords:** undergraduate public health programs, public health education, public health curriculum, BSPH, BAPH, CEPH, ASPPH, United States

## Abstract

**Background:**

Undergraduate programs in public health are becoming increasingly popular in the United States (US). The recent pandemic, growing climate instability, and the aging baby boomers have led to higher demands for skilled public health professionals at various levels of the workforce. This study examines the nature of courses being delivered in undergraduate public health programs across the United States. The goal is to assess domains, themes, competencies, and other specialized skills that are currently covered in these academic programs.

**Methodology:**

A search was conducted in February 2023 using the online CEPH program database to identify undergraduate public health programs in the US. In total, 86 institutions and 90 CEPH-accredited undergraduate public health programs were identified. Lists of public health courses were retrieved from each program, and a total of 2,259 unduplicated courses were extracted and analyzed. A content analysis of the extracted topics was conducted to generate 38 common themes among the courses offered. Coded course themes were mapped to the public health domains and competencies listed by the ASPPH and CEPH to evaluate the distribution of themes across course offerings.

**Results:**

Analysis of course themes found that Foundations of Public Health, Epidemiology, Public Health Management, Policy, and Leadership, Climate and Environmental Health, and Global Health Issues were the most prevalent. When course themes were mapped onto the ASPPH and CEPH domains of critical public health learning, “overview of public health” and “determinants of health” were the most populated domains. Programs had different emphases according to their approach, but overall, about two-thirds of course themes were focused on foundational and theoretical concepts of public health, and one-third were directed toward practical applications of public health concepts.

**Conclusion:**

As the demand for skilled public health workers continues to rise, programs will need to watch the skills and competencies required in the current working environment, as well as the ASPPH and CEPH criteria, and adjust their approach accordingly. Given the rapid changes in the public health landscape, schools and programs of public health should evaluate their curricula to ensure that they are meeting the needs of the workforce and the world.

## 1 Introduction

Public health education has traditionally emphasized a multidisciplinary approach to improving the health and well-being of populations across the globe. In the early 21st century, students in graduate public health programs came from diverse academic backgrounds with little or no public health knowledge (1). However, the upswing in demand for public health workers has made evident the need to provide critical public health skills at the undergraduate level in order to prepare a competent and skilled workforce that can address emerging public health issues (2–5). Public health training offered at the undergraduate level can prepare students to work with communities and other vested partners, including government and non-governmental agencies, in a variety of capacities (3, 6). To identify key issues facing public health undergraduate education, the Association of Schools and Programs of Public Health (ASPPH) established an undergraduate network for public health and global health education for institutions that offer undergraduate majors in public health (7). The Council on Education for Public Health (CEPH) is responsible for accrediting public health education at both the bachelor’s and graduate levels and promotes consistency and quality in curriculum content and academic infrastructure by providing a matrix of subjects and skills that are required to be included in bachelor’s and master’s level public health programs (8).

The common underlying mission among undergraduate public health programs is to ensure that graduating students develop a solid understanding of public health as a discipline and are capable of applying enhanced critical thinking skills to the field of public health (3). To this end, the ASPPH recommended Critical Component Elements (CCEs) for the undergraduate public health curriculum guided the development and adoption of the CEPH guidelines. ASPPH outlines the curricular needs of students in background domains, which aid their comprehension of public health content (9). For example, ASPPH suggests that students have at least foundational knowledge in “Background Domains” of biological and life sciences, social and behavioral sciences, and basic statistics, as well as an introductory experience of the humanities and fine arts. In addition, communication and information literacy skills should be developed among public health undergraduates. ASPPH’s suggested “public health domains” contain nine points: 1. An overview of public health; 2. The role and importance of data in public health; 3. Identifying and addressing population health challenges; 4. Knowledge of human health; 5. Understanding the determinants of health; 6. Knowledge of project implementation; 7. An overview of the health system; 8. Introductory knowledge of health, policy, law, ethics, and economics; and 9. The basics of health communication. The ASPPH also recommended components pertaining to cumulative experiences such as capstone and fieldwork and cross-cutting skills (9).

The CEPH-accreditation criteria for public health undergraduate programs require institutions to provide two foundational competencies: the ability to communicate public health information in both verbal and written form to diverse audiences via a variety of media and the ability to locate, use, evaluate, and synthesize relevant information in 11 foundational domains: (1) Basic statistics; (2) Foundations of biology and life sciences; (3) History, concepts, and values of public health; (4) Methods and tools of data collection; (5) Concepts and approaches to population health; (6) Underlying science of human health; (7) Factors contributing to health disparities; (8) Comprehensive project implementation; (9) US health systems (and others); (10) Legality, ethics, and economics of health care and health policy; (11) Public health communication (10, 11). Programs must also provide opportunities to apply knowledge through experiential activities. Students are required to complete a “capstone” project, which may consist of internships, service-learning projects, portfolio projects, or research projects. CEPH also requires the undergraduate curriculum to include exposure to cross-cutting concepts and experiences that will prepare them to be successful in the workplace or in graduate studies programs. These three criteria, “foundational competencies,” “experiential learning,” and “cross-cutting skills,” provide the framework from which students should be introduced to career trajectories and prepared for graduate studies or professional training in health-related fields (8).

A survey conducted by the Association of American Colleges and Universities (AAC&U) in 2008 showed that 137 (16%) of its 837 members offered majors, minors, or concentrations in public health (12). Tarasenko and Lee’s (13) review of college directories and ASPPH resources revealed that the United States had more than 40 undergraduate programs in public health, community health, or health promotion. The present study is the first of its kind since the COVID-19 pandemic, which served to both clearly highlight the need for skilled public health workers and accelerated demand for public health education (14). It is, therefore, timely to critically assess the nature of public health curriculum at the undergraduate level. This narrative review examines the CEPH-accredited undergraduate public health programs in the US and provides an overview of public health course features. Public health course offerings were assessed for their potential to address the public health domains outlined by both CEPH and ASPPH.

As universities continue to develop undergraduate public health programs (15) and enrolment in public health bachelor’s programs across the US increases (14), a cadre of undergraduates is being prepared to increase the existing public health workforce with strong foundational knowledge in key aspects of public health practice (3, 16). These graduates have the potential to bring a much-needed public health perspective to their respective fields. Building upon the works of Kiviniemi and Przbyla (6) and Osiecki et al. (17), and in consideration of the ASPPH’s Framing The Future 2030 call to action (18, 19) and imminent updates to CEPH’s undergraduate criteria, we carried out an independent, national-level survey of all CEPH-accredited undergraduate public health programs with the goal of investigating the distribution of courses across various domains of public health knowledge and seeking a closer look at how undergraduate health programs are addressing the national standards in public health education and meeting the needs of the students. At the same time, we also intended to assess whether the current public health training is preparing the public health workforce for emerging public health challenges globally. While analyzing the current curricular landscape in undergraduate public health education, we offer insights into the future direction of public health education at the undergraduate level. These insights, matched with ASPPH’s release of the Framing the Future 2030 reports addressing educational transformation in public health (19) could help inform the forthcoming updates to CEPH’s undergraduate criteria in summer 2024.

## 2 Methodology

### 2.1 Data collection

Data collection for this study took place in February 2023 using the search function on the CEPH website to identify accredited institutions offering bachelor’s degrees in public health. Out of 231 programs identified in the initial search, study team members (AJ and SK) filtered programs that offered BA, BS, or BSPH degrees with concentrations of: Community Health, Community Health and Prevention Sciences, Community Health Education, Community Health Practice, Public Health, General Public Health, Health Education, Health Promotion, Health Science, Public Health Science, and Global Health. Programs with concentrations such as Worksite Health Promotion, Wellness, Management, Public Health Sociology, Clinical Health Science, and others with a distinct focus that was not generalized as directly public health-related, were excluded. Institutions that were not in the US were also excluded from this study, resulting in the collection of data from 90 distinct undergraduate public health programs across 86 universities.

### 2.2 Data extraction

**Figure 1:**
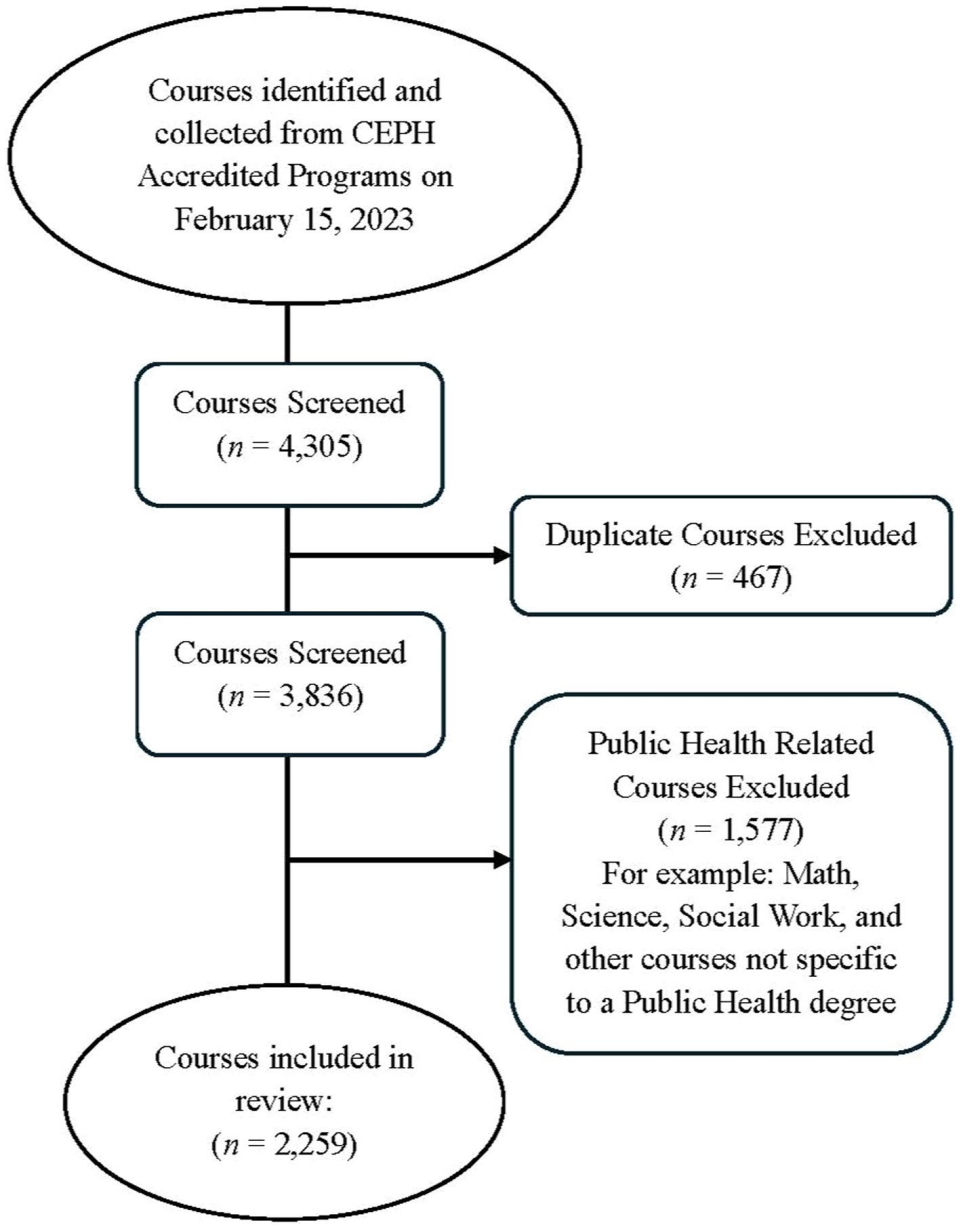
Course data collection flow chart.

Data extracted about institutions included: the name of the institution and program; the city and state where the institute is located; whether the institution is public or private; and whether the program is offered on-campus or online (see Table 1). To gain a clear picture of the range of undergraduate offerings available, we gathered data on the public health courses offered by these undergraduate public health programs. Lists of public health-related courses were extracted from the websites of each selected institution and program, with 4,305 courses initially collected and screened. Duplicate courses were identified first within individual institutional listings by course number, then by verification of a duplicated course name (*n* = 469). The remaining courses (*n* = 3,836) were screened, and non-public health-related courses were excluded (*n* = 1,577). Courses excluded in this round included general education courses that were not directly related to public health (e.g., mathematics, chemistry, physics, or other science and humanity courses), and elective courses from departments other than public health (e.g., social work, sociology, anthropology, etc.). After these two rounds of exclusion, a total of 2,259 courses remained. A detailed step-by-step search strategy for the entire process is presented in the flowchart (Figure 1).

**Table 1:** Institution distribution (*n* = 90).

Region	Private	Public	Total institutions	States represented
Northeast	9	14	23	Connecticut, Maine, Maryland, Massachusetts, New Jersey, New York, Pennsylvania, Rhode Island, Vermont
Southeast	4	25	29	Florida, Georgia, Kentucky, Louisiana, Mississippi, North Carolina, South Carolina, Tennessee, West Virginia, Washington DC
Midwest	4	15	19	Illinois, Indiana, Iowa, Kansas, Michigan, Minnesota, Missouri, Nebraska, Oklahoma, Wisconsin
Southwest	2	2	4	Arizona, Texas
West	4	11	15	California, Hawai’i, Montana, Nevada, Oregon, Utah
**Total**	23	67	90	37 states

### 2.3 Data analysis

We carried out a descriptive analysis of the data elements collected. The locations of the institutions were categorized into five geographical regions consistent with the US Census Bureau division, with an additional delineation of the western states: Northeast, Southeast, Midwest, Southwest, and West. Institutional status as public or private was identified. The frequency of on-campus versus hybrid on-campus/online was noted. Course titles (*n* = 2,259) were coded into subject themes. These coded themes were then analyzed for relevance to an integrated matrix of the ASPPH’s recommended Public Health Domains and Cumulative Field Exposure elements alongside the CEPH Foundational Domains and Cumulative and Experiential Activities for an undergraduate public health major. In case the coding placement of a course was in question, co-authors investigated the nature of the placement of the course within the curriculum and discussed it among themselves to reach a consensus for the appropriate placement.

## 3 Results

### 3.1 Institutional analysis

Based on the CEPH Degree Program Search, 90 undergraduate public health programs are offered by 86 institutions across the US. Of the 90 undergraduate public health programs, 74% (*n* = 67) are offered by public institutions, and 26% (*n* = 23) are offered by private institutions. Undergraduate public health programs are distributed across 37 states, with the majority (*n* = 29) in the Southeast, followed by the Northeast (*n* = 23), and then the Midwest (*n* = 19). Only four (*n* = 4) programs were identified in the Southwest, and 15 accredited programs were found in the West. The northeast has the highest number of private programs (*n* = 9). There are fewer private programs in the Southeast, Midwest, and West (4 programs each), but the proportion of private programs is lowest in the Southwest (*n* = 2) (see Table 1). No CEPH-accredited school or program offers a completely online undergraduate public health program, but 47% offer a hybrid on-campus/online program.

### 3.2 Thematic course analysis

Among the 2,259 public health courses, 38 themes were derived based on the similarity in their titles and inferred course content (see Table 2; Appendix A). The frequencies of each coded course theme are calculated based on the number of individual courses associated with the theme.

**Table 2:** Distribution of subject themes of courses (*n* = 2,259).

Coded subject themes of courses	*n*
Foundations of Public Health	148
Epidemiology	135
Public Health Management, Policy, and Leadership	120
Climate and Environmental Health	116
Global Health Issues	116
Social Determinants and Health Disparities	113
Population Health Program Design & Implementation	91
Health Education, Promotion, and Advocacy	89
Public Health Research Methods	83
Health Behavior Theory and Practice	80
Health, Gender, and the Human Lifespan	79
US Health Systems and Policy Issues	77
Independent Study, Internships, and Study Abroad	75
Health Data Analytics and Informatics	68
Biostatistics	60
Personal Health and Wellness	60
Capstone Experience	54
Occupational Health and Safety	54
Emergency Preparedness and Disaster Management	53
Chronic Disease and Infectious Disease	49
Substance Use, Addiction, and Violence Prevention	48
Fieldwork/Practicum	44
Community Nutrition	41
Biological Basis of Disease	37
Health Communication	36
Public Health Economics and Finance	35
Food and Water Access and Safety	34
Sequential academic preparation courses	34
Special Topics and Populations	33
Sexual Health and Reproductive Issues	33
Population Health Intervention and Evaluation	28
Health Sciences	28
Law/Ethics in Healthcare	23
Mental Health	20
Technical Writing for Public Health	19
History of Public Health	16
Community Health in Schools and Colleges	16
Media and Marketing	14

#### 3.2.1 Course theme frequencies >100

Six themes have frequencies greater than 100. Foundations of Public Health (*n* = 148), which includes courses related to foundational principles of public health; community health; and population health, is the most popular course theme, followed by Epidemiology (*n* = 135). The three themes that tied with *n* = 120 are Public Health Management, Policy, and Leadership; Climate and Environmental Health; and Global Health Issues. The theme of Social Determinants and Health Disparities (*n* = 113), which encompasses courses about social justice, health equity, and human rights as well as the social determinants of health and disease, is another popular subject in undergraduate curriculum.

#### 3.2.2 Course theme frequencies between 75 and 100

Seven themes are identified in this category of courses. Population Health Program Design and Implementation (*n* = 91) and Health Education, Promotion, and Advocacy (*n* = 89) are foremost in this category. Public Health Research Methods (*n* = 83) follows, which includes training in quantitative and qualitative methods, participatory research, demographic analysis, and clinical trials. Health Behavior Theory and Practice (*n* = 83), the extensive topic dealing with Health, Gender, and the Human Lifespan (*n* = 79), and US Health Systems and Policy Issues (*n* = 77), which includes courses examining the healthcare systems in the US and comparing them with others, also fall into this category. Finally, Independent Study, Internships, and Study Abroad courses are identified 75 times.

#### 3.2.3 Course theme frequencies between 50 and 75

The rapidly growing field of Health Data Analytics and Analysis is mentioned 68 times in the course list, including references to health surveillance, medical informatics, longitudinal analysis, and data modelling. The theme of Biostatistics captured courses in applied math and statistics as well as biostatistical courses at the undergraduate level (*n* = 60). Personal Health and Wellness courses (*n* = 60) pertain to topics of fitness and personal health, including physical activity, healthy living, and stress management. The Capstone Experience (*n* = 54) is a key component of most undergraduate public health programs, which was categorized based on phrases such as “capstone” and “culminating experience.” Occupational Health and Safety is the topic of 54 courses, followed by Emergency Preparedness and Disaster Management (*n* = 53), which includes courses covering states of emergency, risk assessment, and emergency response.

#### 3.2.4 Course theme frequencies between 25 and 50

As the frequencies decrease, the diversity of themes increases. Thirteen themes are included in this subgroup, including the broad category of Chronic and Infectious Disease (*n* = 49), which addresses disease management, prevention, and control, emergent threats, COVID-19, and HIV/AIDS, and the course theme of Substance Use, Addiction, and Violence Prevention (*n* = 48), which addresses addiction to alcohol, tobacco, opioids, and other substances. Fieldwork/practicum studies (*n* = 44) could be grouped with the variety of subtopics addressed by each of the studies but are maintained as a separate theme due to the distinction of usually being networked with community agencies beyond the campus. Other subjects in this subgroup include Community Nutrition (*n* = 41); the Biological Basis of Disease (*n* = 37); Health Communication (*n* = 36); Public Health Economics and Finance (*n* = 35); and Food and Water Access and Safety (*n* = 34). There are 34 mentions of institution-specific sequential academic preparation courses, such as “The First Year Experience,” “Public Health Senior Seminar,” and “Thesis Research.” The theme of Special Topics and Populations (*n* = 33) contains a wide variety of regional and population-specific public health subjects, including indigenous issues, rural health, disabilities and public health, and other special topics. Sexual Health and Reproductive Issues (*n* = 33), Population Health Intervention and Evaluation (*n* = 28), which is a subset of Program Design and Implementation but has been given its own theme to address the variety of assessment and evaluation methods, and Health Sciences (*n* = 28), including medical terminology and studies of various health modalities.

#### 3.2.5 Course theme frequencies <25

The six course themes in this class of courses include Law/Ethics in Healthcare (*n* = 23) and issues around Mental Health (*n* = 20), as well as Technical Writing for Public Health (*n* = 19), which encompasses technical writing, such as for grants and proposals, as well as other academic and scientific writing. Courses specifically pertaining to the History of Public Health comprise 16 instances in the data, as do courses about Community Health in Schools and Colleges (*n* = 16), which cover community health in academic institutions from elementary through graduate school. The specialized topic of Media and Marketing (*n* = 14) includes courses that review the presentation of public health information across various media and skills for marketing public health programs via social marketing and other forms of mass media.

### 3.3 Analysis of course themes within foundational domains of public health

To gain a better picture of the structure of the undergraduate public health curriculum in relation to the ASPPH CCEs and CEPH requirements, CEPH’s 11 Foundational Domains and ASPPH’s nine Public Health Domains were overlaid. CEPH’s Foundational Domains One (concepts and applications of basic statistics) and Three (history and philosophy of public health) are combined and grouped with ASPPH’s Public Health Domain One (Overview of Public Health), and CEPH’s Foundational Domains Two (the foundations of biological and life sciences) and Six (underlying science of human health and disease) are combined and grouped with ASPPH’s Public Health Domain Four (Human Health). For the sake of brevity, the 38 coded course themes are mapped onto the nine domains and two cumulative experience requirements in ASPPH’s Public Health Domains and Cumulative Experience recommendations (see Table 3; Appendix B). Requirements for cumulative experience and field exposure are concurrent.

**Table 3:** Course frequencies mapped into ASPPH and CEPH foundational domains.

ASPPH Recommended Public Health Domains for undergraduate public health education	CEPH Public Health bachelor’s degree Foundational Domains criteria for undergraduate public health education	Frequency (*n* = 2,259)	Percent (%)
1. Overview of Public Health	1. Concepts and applications of basic statistics 3. The history and philosophy of public health as well as its core values, concepts, and functions across the globe and in society	509	22.5%
2. Roles and Importance of Data in Public Health	4. The basic concepts, methods, and tools of public health data collection, use, and analysis and why evidence-based approaches are an essential part of public health practice	151	6.7%
3. Identifying and Addressing Population Health Challenges	5. The concepts of population health, and the basic processes, approaches and interventions that identify and address the major health-related needs and concerns of populations	114	5.0%
4. Human Health	2. Foundations of biological/life science 6. The science of human health and disease, including opportunities for promoting and protecting health across the life course	294	13.0%
5. Determinants of Health	7. The socioeconomic, behavioral, biological, environmental, and other factors that impact human health and contribute to health disparities	330	14.6%
6. Project Implementation	8. The fundamental concepts of project implementation, including planning, assessment, and evaluation	135	6.0%
7. Overview of the Health System	9. The fundamental characteristics and organizational structures of the US health system and differences between systems in other countries	77	3.4%
8. Health Policy, Law, Ethics, and Economics	10. Basic concepts of legal, ethical, economic, and regulatory dimensions of health care and public health policy and the roles, influences, and responsibilities of the different agencies and branches of government	285	12.6%
9. Health Communication	11. Concepts of public health-specific communication, including technical and professional writing and the use of mass media and electronic technology	158	7.0%

ASPPH Cumulative Experience and Field Exposure	CEPH Public Health bachelor’s degree Cumulative & Experiential Activities		
1. Cumulative Experience	(CEPH does not divide this category any further)	87	3.9%
2. Field Experience		119	5.3%

#### 3.3.1 ASPPH domain 1. Overview of public health

##### CEPH foundational domains 1 and 3

The first domain includes coded themes considered foundational to all studies of public health, such as: Foundations of Public Health (*n* = 148), History of Public Health (*n* = 16), Biostatistics (*n* = 60), Epidemiology (*n* = 135), Sequential academic preparation courses (*n* = 34), and Global Health Issues (*n* = 116). The total frequency of courses sorted into this domain is 509, the highest of all the domains.

#### 3.3.2 ASPPH domain 2. Roles and importance of data in public health

##### CEPH foundational domain 4

The second domain contains two of the course theme categories: Health Data Analytics and Informatics (*n* = 68) and Public Health Research Methods (*n* = 83), with a total frequency of 151 courses.

#### 3.3.3 ASPPH domain 3. Identifying and addressing population health challenges

##### CEPH foundational domain 5

The third domain contains the themes of Health Behavior Theory and Practice (*n* = 80) and Food and Water Access and Safety (*n* = 34), representing 114 courses.

#### 3.3.4 ASPPH domain 4. Human health

##### CEPH foundational domains 2 and 6

The fourth ASPPH domain covers a broad range of information. Six course themes are included in this category: Chronic Disease and Infectious Disease (*n* = 49); Personal Health and Wellness (*n* = 60); Health, Gender, and the Human Lifespan (*n* = 79); Health Sciences (*n* = 28); and Community Nutrition (*n* = 41). Unduplicated courses associated with this domain total 294.

#### 3.3.5 ASPPH domain 5. Determinants of health

##### CEPH foundational domain 7

Determinants of Health is one of the most discussed components of public health in recent years and the second most popular course offering. Five course themes are grouped in this domain, and two of them, Social Determinants and Health Disparities (*n* = 113) and Climate and Environmental Health (*n* = 116) are responsible for most of the courses sorted therein. The other three themes: Substance Use, Addiction, and Violence Prevention (*n* = 48), Sexual Health and Reproductive Issues (*n* = 33), and Mental Health issues (*n* = 20) account for about one third of the total frequency for this section (*n* = 330).

#### 3.3.6 ASPPH domain 6. Project implementation

##### CEPH foundational domain 8

Three themes represent the course content pertaining to the subject area. Courses grouped with Population Health Program Design & Implementation (*n* = 91), Population Health Intervention and Evaluation (*n* = 28), and Community Health in Schools and Colleges (*n* = 16) represent a total frequency of 135.

#### 3.3.7 ASPPH domain 7. Overview of the health system

##### CEPH foundational domain 9

The only course theme for the seventh and smallest domain is US Health Systems and Policy Issues (*n* = 77).

#### 3.3.8 ASPPH domain 8. Health policy, law, ethics, and economics

##### CEPH foundational domain 10

The eighth public health domain includes an extensive course of study and thus contains an array of five course themes: Public Health Management, Policy, and Leadership (*n* = 120); Law/Ethics in Healthcare (*n* = 23); Public Health Economics and Finance (*n* = 35); Occupational Health and Safety (54); and Emergency Preparedness and Disaster Management (*n* = 53), with a total frequency of 285.

#### 3.3.9 ASPPH domain 9. Health communication

##### CEPH foundational domain 11

The ninth domain is represented by four course themes. The broad theme of Health Education, Promotion, and Advocacy (*n* = 89) comprises the bulk of the domain. However, the remaining three themes, though not highly populated, are still critical to a modern public health education: Technical Writing for Public Health (*n* = 19), Media and Marketing (*n* = 14), and Health Communication (*n* = 36). The total number of courses designated to this domain was 158.

#### 3.3.10 ASPPH cumulative experience and field exposure

##### CEPH cumulative and experiential activities

The ASPPH guidelines for Cumulative Experience and Field Exposure contain two sections: Cumulative Experience and Field Experience. CEPH requires “Cumulative and Experiential Activities” but does not categorize them further. The domain of cumulative experience carries the course themes for Capstone Experience (*n* = 54), and Special Topics and Populations (*n* = 33), for a total frequency of 87. The Field Experience domain (*n* = 119) also contains only two themes: Independent Study, Internships, and Study Abroad (*n* = 75), and Fieldwork/Practicum (*n* = 44). For a concise representation of domains and frequencies mapped onto the ASPPH Domains, see Table 3.

## 4 Discussion

The recent global experience of the COVID-19 pandemic, in conjunction with the increasing multi-faceted threats of climate change and recent reckonings with widespread social, racial, and economic iniquities, is leading to a greater need for and recognition of the importance of public health as a basic social good (16, 20, 21). This study explores the current trends in undergraduate health education and assesses the efforts being made to prepare a well-trained public health workforce to meet future demands. The WHO estimates that the world will need 18 million additional health workers by 2030 (16), and national estimates predict job growth in public health fields ranging from 5 to 30% in the next 10 years (22). Further, the most recent national and international public health initiatives, Public Health 3.0 (spearheaded by the US Department of Health and Human Services) and the World Health Organization’s One Health Initiative (23–25), both recommend increased collaboration among diverse entities and peers; engagement with these strategies will lead to more responsive and locally relevant public health initiatives. As such, it behooves academic institutions to provide diverse curricular options for students to engage in more than a narrowly focused field of study. There is an opportunity in undergraduate studies to expose students to the broad range of information that they will need to be able to either work within multilevel collaborations described in the Public Health 3.0 and One Health approaches (6, 17) or pursue higher education in specialized public health and other health-related fields.

This study investigates the curricula of 90 undergraduate public health programs offered by 86 institutions in 37 US states, as identified through CEPH’s program search engine. Analysis at the institutional level revealed that three-quarters (74%) of undergraduate programs were offered by public institutions. This aligns with previously published literature, which reported that undergraduate public health programs are predominantly offered at public universities (8). Geographical distributions of the programs showed the highest frequency in the Southeast region (32%), followed closely by the Northeast (26%). The West (17%) and Southwest (4%) were the least represented. These figures are reflective of regional population density and development. Public health programs in private institutions were more prevalent in the Northeast, where 39% of the region’s institutions are private and there is a greater concentration of wealth. The Southeast has the fewest private institutions offering public health undergraduate degrees (13.8%). The number of CEPH-accredited institutions offering undergraduate public health programs has greatly increased, from 13 reported in 2005 to 86 identified in this study (26).

An analysis of course themes reveals that the primary areas of public health studies are being broadly addressed at the undergraduate level. When course themes are mapped onto CEPH’s Foundational Domains and the recommended Public Health Domains and Cumulative Experience and Field Exposure competencies of the ASPPH, the trends in undergraduate public health programs become clearer. The first domain, Overview of Public Health, which includes introductions to all the core public health disciplines, distinctly contained 22.5% of the analyzed courses. Three domains: Determinants of Health (including social and environmental determinants and discussions of equity, justice, and related issues), Human Health, and Health Policy, Law, Ethics, and Economics, are a cluster represented by 12 to 15% of courses. These distributions are proportional and reasonable given the breadth of information covered.

However, another cluster of four domains: Roles and Importance of Data in Public Health, Identifying and Addressing Population Health Challenges, Project Implementation, and Health Communication, are all represented by just 5 to 7% of courses. The low percentage in this domain is concerning given the global challenge in the public health sector to address misinformation regarding COVID-19 prevention, vaccination, and other health issues of great concern (27–29). The future public health workforce will need to be well-trained in translating scientific information into forms that can be communicated to lay community members in a variety of mediums and formats. The other outlying public health domain was Overview of the Health System, with only 3.4% of the total analyzed course data. There were slightly more course listings for Field Experience (5.4%) than Cumulative Experience (3.9%), but combined, the category for experiential and field-based learning represents just 9.2% of the total curriculum.

This analysis indicates that undergraduate public health programs are structured to cover a broad swath of subject matter and give students both a knowledge and experiential base from which to grow into their chosen workplace or continue further study in a specialized field. An examination of course theme distribution across the CEPH and ASPPH’s domains shows that undergraduate schools and programs are aligned with the liberal education approach (30) and thus are focused more on foundational information than skills for direct application in public health practice, as the most populated domains tended to be theoretical and conceptual aspects. In total, the domains and accompanying themes pertaining to the application of public health (Cumulative and Field Experience, Health Communication, Project Implementation, Addressing Population Health Challenges, and Roles and Importance of Data in Public Health) account for about one third (33.9%) of the total curriculum, which, considering the breadth of interdisciplinary subjects that must be covered during a well-rounded undergraduate program, may be an appropriate balance. As argued by Kiviniemi and Makenzie (30), this liberal education approach is appropriate for undergraduate public education due to its capacity to provide problem-solving and critical thinking skills that will serve students in any professional setting, as well as the necessary theoretical foundation in public health methods.

While there are no recent studies that examine the public health undergraduate curriculum at the national level in the US, the curricular field has greatly expanded in recent years. A few other studies of specific programs’ curricula provide excellent examples of curriculum development as well as student outcomes, which aided our process of interpreting the outcomes of this study (6, 12, 31, 32). Resnick et al.’s (7) thorough qualitative examination of undergraduate public health makes a useful companion for consideration of the issues at hand for both public health higher education and the future of the public health workforce.

As the importance of public health increases and opportunities for public health education expand, it will be all the more important to maintain a foundation of evidence-based information to support the framework of the public health curriculum (33). It is not enough to know that the accreditation criteria are being fulfilled; we must continue to examine the quality of how they are being addressed and which elements are being emphasized (34). In order to address the changing needs of the world and to thrive in the modern workplace, students must be trained in skills such as effective public health communication, data and information science, and financial management, as well as receive foundational knowledge of classic public health disciplines (35–37). Our findings illustrate the distribution of courses in public health undergraduate education nationwide, how theoretical and practical skills are represented and balanced with cross-cutting experiences, and field experience opportunities.

Further, as part of the recommendations derived from this study, we suggest that undergraduate programs in public health offer an option for students to take pre-med courses as part of their general education, including biology, chemistry, physics, and psychology. Public health as a pre-med major could create a new cadre of clinicians who understand community health, making them more effective practitioners. We also recommend greater emphasis on themes around adolescent health such as sexual reproductive health, non-communicable diseases, injuries and violence, nutrition, mental health, and substance abuse as integral parts of all curricula, or at least offer these population-specific topics as suggested elective components. This will serve two purposes: one, it will be more attractive, relevant, and applicable to undergraduate students; and two, adolescent health expertise is much needed as adolescents constitute close to 1.2 billion (16%) of the total world population, and their health is central to the growth and development of any nation. Undergraduate public health education may also be critical to the preparation of a public health workforce that can respond to rapidly evolving health dynamics and disease patterns linked to climate change, population expansion, migration, and managing environmental crises on the horizon. Addressing these rapidly evolving health dynamics will demand enhancements in curricular content, including a stronger emphasis on skill-based and experiential training in emergency preparedness and effective response.

### 4.1 Limitations and strengths

This study is limited to a survey of US institutions and programs. Also, it does not account for whether programs offer both majors and minors and how long the undergraduate program has been in existence. We did not analyze the general education courses or elective courses, which are integral to undergraduate education. We captured course data based on the prefix listed for a course, and analysis is based on the course name, which required interpretation as each institute had its own system of course code delineation. It is possible that some public health courses were excluded, overlooked, or omitted due to this method of data capture. Analysis based solely on the course name is also limiting. Access to the syllabi would have provided much greater detail about the specific nature of each course’s content and may have affected our interpretation and coding. We hope that this study will inspire further investigations into the detailed structure, syllabi, and pedagogical approaches to undergraduate public health education.

Since each of the programs included in this study is CEPH accredited, they are, by necessity, fulfilling CEPH accreditation requirements. The methods of data collection and analysis are not designed to capture the strengths of each program but instead to provide a sweeping narrative review. Due to the above-mentioned limitations in the nature of the data collected, we did not analyze course themes pertaining to CEPH’s Foundational Competencies, which are called “Background Domains” by ASPPH and include the synthesis of public health information and the ability to communicate public health information, as well as areas of general education. We also considered the analysis of cross-cutting concepts and areas within the ASPPH and CEPH frameworks outside the scope of this study, as course titles and themes alone cannot give enough information about the nature of each course to evaluate its relevance in those areas.

Despite the limitations above, the study has strengths in that an extensive review and comprehensive analysis were conducted using the census of all CEPH-accredited public health undergraduate programs. The study also assesses the courses offered based on the standards of two national educational/accrediting boards (ASPPH and CEPH). The study is timely because it provides critical information about our public health academic programs for training the entry-level public health workforce post-COVID-19 pandemic through the lens of lessons learned and gaps and inequalities in the public health and healthcare delivery systems exposed by the pandemic.

### 4.2 Public health implications

It is imperative to assess the landscape of course offerings in undergraduate public health programs to better gauge the skills, knowledge, and training being provided, especially in the context of the CEPH and ASPPH frameworks. Our intent is that this study will contribute to the current knowledge base of undergraduate public health and assist programs as they consider their curriculum development and revisions. Studies of this nature will be critical as academic institutions continue to modify their curricula to adequately prepare students for the rapidly evolving demands of the 21st-century public health workforce.

## References

[1] Riegelman RK Albertine S. Undergraduate public health at 4-year institutions: It’s here to stay. Am J Prev Med. (2011) 40:226–31. doi: 10.1016/j.amepre.2010.10.013 21238873

[2] Baker PRA Dingle K Dunne MP. Future of public health training: what are the challenges? What might the solutions look like? Asia Pac J Public Health. (2018) 30:691–8. doi: 10.1177/1010539518810555, PMID: 30444136

[3] Bass SB Guttmacher S Nezami E. Who will keep the public healthy? The case for undergraduate public health education: a review of three programs. J Public Health Manag Pract. (2008) 14:6–14. doi: 10.1097/01.PHH.0000303407.81732.02, PMID: 18091034

[4] Gerberding JL. Back to the future of public health. Am J Public Health. (2021) 111:596–7. doi: 10.2105/AJPH.2021.306176, PMID: 33689423 PMC7958022

[5] Institute of Medicine (US) In: Gebbie K Rosenstock L Hernandez LM, editors. Who will keep the public healthy? Educating public health professionals for the 21st century: National Academies Press (US) (2003) Available at: http://www.ncbi.nlm.nih.gov/books/NBK221182/ (Accessed November 2, 2022). 25057636

[6] Kiviniemi MT Przybyla SM. Integrative approaches to the undergraduate public health major curriculum: strengths, challenges, and examples. Front Public Health. (2019) 7:106. doi: 10.3389/fpubh.2019.00106, PMID: 31114779 PMC6503149

[7] Resnick B Selig S Riegelman R. An examination of the growing US undergraduate public health movement. Public Health Rev. (2017) 38:4. doi: 10.1186/s40985-016-0048-x 29450076 PMC5809906

[8] Resnick B Leider JP Riegelman R. The landscape of US undergraduate public health education. Public Health Rep. (2018) 133:619–28. doi: 10.1177/0033354918784911, PMID: 30084738 PMC6134569

[9] Wykoff R Petersen D Weist EM. On academics: the recommended critical component elements of an undergraduate major in public health. Public Health Rep. (2013) 128:421–4. doi: 10.1177/003335491312800516, PMID: 23997294 PMC3743296

[10] CEPH. Accreditation criteria for schools of public health and public health programs Council on Education for Public Health (2021).

[11] Chorazy ML Klinedinst KS. Learn by doing: a model for incorporating high-impact experiential learning into an undergraduate public health curriculum. Front Public Health. (2019) 7:31. doi: 10.3389/fpubh.2019.00031, PMID: 30863743 PMC6399422

[12] Gebo KA Goodyear JD David SR Yager JD. Public health studies as an undergraduate major. Public Health Rep. (2008) 123:812–7. doi: 10.1177/003335490812300620, PMID: 19711664 PMC2556709

[13] Tarasenko YN Lee JM. U.S. undergraduate education in public health: hot or not? Front Public Health. (2015) 3:71. doi: 10.3389/fpubh.2015.00071, PMID: 26029686 PMC4426683

[14] ASPPH. (2021). 2021 annual report. Available at: https://aspph.org/about/annual-report-pages/annual-report-2021/ (Accessed December 11, 2022).

[15] Nelson-Hurwitz DC Tagorda M Kehl L Buchthal OV Braun KL. Developing an undergraduate public health introductory Core course series. Front Public Health. (2018) 6:155. doi: 10.3389/fpubh.2018.00155, PMID: 29892596 PMC5985697

[16] Bashier H Ikram A Khan MA Baig M Gunaid MA Nsour MA. The anticipated future of public health services post COVID-19: viewpoint. JMIR Public Health Surveill. (2021) 7:e26267. doi: 10.2196/26267, PMID: 33592576 PMC8216329

[17] Osiecki K Barnett J Mejia A. Creating an integrated undergraduate public health curricula: inspiring the next generation to solve complex public health issues. Front Public Health. (2022) 10:864891. doi: 10.3389/fpubh.2022.864891, PMID: 35509505 PMC9059934

[18] ASPPH. Framing the future Association of Schools and Programs of Public Health (ASPPH) (2024) Available at: https://aspph.org/our-work/initiatives/framing-the-future/ (Accessed March 12, 2024).

[19] Sullivan LM Weist EM Barrington WE Hwang W Kiviniemi MT Mohammed SD. Education for public health 2030: transformation to meet health needs in a changing world. Front Public Health. (2023) 11:1269272. doi: 10.3389/fpubh.2023.1269272 38162596 PMC10757328

[20] Krasna H Czabanowska K Jiang S Khadka S Morita H Kornfeld J. The future of careers at the intersection of climate change and public health: what can job postings and an employer survey tell us? Int J Environ Res Public Health. (2020) 17:1310. doi: 10.3390/ijerph17041310, PMID: 32085475 PMC7068354

[21] Leitch S Corbin JH Boston-Fisher N Ayele C Delobelle P Gwanzura Ottemöller F. Black lives matter in health promotion: moving from unspoken to outspoken. Health Promot Int. (2020) 36:1160–9. doi: 10.1093/heapro/daaa121, PMID: 33305322 PMC7953963

[22] Advocates for Public Health Education. (2022). 25+ Most in-demand Public Health Jobs & Titles *|* *MPHProgramsList.com* *2022* [MPHProgramsList com 2022 |]. https://Mphprogramslist.Com. Available at: https://mphprogramslist.com/25-public-health-jobs-most-in-demand/ (Accessed October 14, 2022).

[23] One Health High-Level Expert Panel Adisasmito WB Almuhairi S Behravesh CB Bilivogui P Bukachi SA. One health: a new definition for a sustainable and healthy future. PLoS Pathog. (2022) 18:e1010537. doi: 10.1371/journal.ppat.1010537, PMID: 35737670 PMC9223325

[24] Rosa WE Hassmiller SB. The sustainable development goals and building a culture of health. Am J Nurs. (2020) 120:69–71. doi: 10.1097/01.NAJ.0000668772.33792.1f 32443132

[25] UNDP. Sustainable development goals UNDP (2022) Available at: https://www.undp.org/sustainable-development-goals (Accessed November 6, 2022).

[26] Associations of Schools of Public Health. Schools of public health survey 2005 Associations of Schools of Public Health (2005) Available at: http://www.asph.org/document.cfm?page=978 (Accessed November 6, 2022).

[27] Kirk Sell T. Meeting COVID-19 misinformation and disinformation head-on John Hopkins Bloomberg School of Public Health (2023) Available at: https://publichealth.jhu.edu/meeting-covid-19-misinformation-and-disinformation-head-on (Accessed July 27, 2023).

[28] Lee SK Sun J Jang S Connelly S. Misinformation of COVID-19 vaccines and vaccine hesitancy. Sci Rep. (2022) 12:Article 1. doi: 10.1038/s41598-022-17430-6, PMID: 35953500 PMC9366757

[29] Lopes L Stokes M 2021. KFF COVID-19 vaccine monitor: media and misinformation KFF (2021) Available at: https://www.kff.org/coronavirus-covid-19/poll-finding/kff-covid-19-vaccine-monitor-media-and-misinformation/ (Accessed July 27, 2023).

[30] Kiviniemi MT Mackenzie SLC. Framing undergraduate public health education as Liberal education: who are we training our students to be and how do we do that? Front Public Health. (2017) 5:9. doi: 10.3389/fpubh.2017.00009, PMID: 28239603 PMC5301016

[31] Armstrong-Mensah EA Ramsey-White K Alema-Mensah E Yankey BA. Preparing students for the public health workforce: the role of effective high-impact educational practices in undergraduate public health program curricula. Front Public Health. (2022) 10:790406. doi: 10.3389/fpubh.2022.790406, PMID: 35400063 PMC8987350

[32] Nelson-Hurwitz DC Tagorda M Kehl L Patil U. What can you do with a Bachelor’s in public health? A case study of graduate outcomes from the University of Hawai‘i. Front Public Health. (2021) 9:661629. doi: 10.3389/fpubh.2021.661629, PMID: 34434912 PMC8380951

[33] Petersen DJ Weist EM. Framing the future by mastering the new public health. J Public Health Manag Pract. (2014) 20:371–4. doi: 10.1097/PHH.0000000000000106 24813674 PMC4032212

[34] Godley S Aumiller B Horigian V Khalil N Kruger J Pennel C. Evidence-based educational practices for public health: how we teach matters. Pedagogy Health Promot. (2021) 7:89–94. doi: 10.1177/2373379920978421

[35] Goldsmith J Sun Y Fried LP Wing J Miller GW Berhane K. The emergence and future of public health data science. Public Health Rev. (2021) 42:1604023. doi: 10.3389/phrs.2021.1604023 34692178 PMC8378512

[36] Joshi A Gertner R Roberts L El-Mohandes A. An evidence-based approach on academic Management in a School of public health using SMAART model. Sustainability. (2021) 13:Article 21. doi: 10.3390/su132112256

[37] Resnick BA Morlock L Diener-West M Stuart EA Spencer M Sharfstein JM. PH WINS and the future of public health education. J Public Health Manag Pract. (2019) 25:S10–2. doi: 10.1097/PHH.0000000000000955, PMID: 30720612 PMC6519884

