# Supplementary material for: A narrative review of the CEPH-accredited bachelor’s public health programs’ curricula in the United States
Source: Front Public Health. 2024 Aug 21;12:1436386. doi: 10.3389/fpubh.2024.1436386 (PMC11371682; doi:10.3389/fpubh.2024.1436386)
Supplement: Supplementary file 1 [file Table_1.docx]

**Supplementary Materials**

**Appendix A. Coded Course Frequencies, Themes, and Examples (*n* = 2,259)**

| **Freq**  ***n*** | **Coded Course Themes** | **Examples of specific course titles included in the theme** |
| --- | --- | --- |
| 148 | Foundations of Public Health | Introduction to Public Health, Community Health, Public Health Foundations, Principles of Public Health, Fundamentals of Health, Public Health Concepts, Public Health in Action, Public and Community Health, Introduction to Community Health |
| 135 | Epidemiology | Epidemiology, Applied Epidemiology, Concepts in Epidemiology, Disease Detectives: Epidemiology, Epidemiological Study and Analysis, Epidemiology: The Science of Public Health, Human Disease and Epidemiology, Principles of Epidemiology, Pharmacoepidemiology, Neuroepidemiology |
| 120 | Public Health Management, Policy, and Leadership | Health care policy, Policy and Management, Policy and Advocacy, Policy and Systems, Law Policy and Justice, Policy Analysis, Policy and Governance, Policy and Systems of Power |
| 116 | Climate and Environmental Health | Climate change and health, Environmental Determinants of Health, Environmental Health-Urban, Built Environment, Environmental Policy, Environmental Justice and Ethics, Sustainability, Geology of Health, Environmental Health in the Developing World, GIS Public Health Applications |
| 116 | Global Health Issues | Global environmental health, Global Nutrition, One Health, Global Health and Security, Global Health Policy, Global Development, Global communicable disease, Global Equity, Global Health Interventions, Global Health Service Delivery, Global Immigration and Refugee Health, Global Infectious Disease, Global Road Safety |
| 113 | Social Determinants and Health Disparities | Social Determinants of Health, Poverty and Health, Determinants of Disease, Cultural Competency, Health Equity, Social Justice, Global Urban Equity, Human Rights, Mass Incarceration, Social Action, Social and Ecological Determinants of Health |
| 91 | Population Health Program Design & Implementation | Public Health Planning, Health Program Implementation, Public Health Practice, Public Health Interventions, Health Promotion Planning, Public Health Project Implementation, Project Design and Implementation, Public Health Intervention Design |
| 89 | Health Education, Promotion, and Advocacy | Public Health Education, Advocacy in Public Health, Public Health Promotion and Outreach, Health Education and Health Promotion, Peer Education, Public Health Educational Planning, Public Health Program Promotion, Making a Difference with Public Health Advocacy |
| 83 | Public Health Research Methods | Participatory Research, Quantitative methods, Qualitative methods, Research Ethics, Clinical Methods, Indigenous Research Methods, Clinical Trials Management, Demographic Analysis, Introduction to Clinical Trials |
| 80 | Health Behavior Theory and Practice | Health Behavior, Obesity, Behavior Change Theories, Behavior Change and Technology, Behavioral Change Foundations, Behavioral Health Methods, Health Behavior Harm Reduction, Health in Society, Homelessness and Stigma, Trauma, Abuse, Social Context of Public Health |
| 79 | Health, Gender, and the Human Lifespan | Women's Health, Men's Health, Maternal/Child, LGBTQ+ Health, Family Health, Death and Dying, Health in Later Years, Health Aspects of Aging |
| 77 | US Health Systems and Policy Issues | US Public health care system, Comparative health care systems, Health care systems around the world, Health Systems, Introduction to health Systems, The US Healthcare System, Politics and Payments in US Healthcare, Healthcare Delivery |
| 75 | Independent Study, Internships, and Study Abroad | Public Health Internship, Internship in Health Data Analytics, Epidemiology Internship, Global Health Internship, Mentored Research, Independent Study in Health & Safety, Global Health Immersion: Latin America, Undergraduate Public Health Study Abroad |
| 68 | Health Data Analytics and Informatics | Data Modelling for Public Health, Data Analysis, Introduction to Healthcare Informatics, Health Informatics, Health Surveillance, Healthcare Information Systems, Medical Informatics, Longitudinal Analysis, Public Health Informatics |
| 60 | Biostatistics | Biostatistics I & II, Biostatistics and Epidemiological Computing, Biostatistical Research Methods, Statistics, Biostatistics for Health Science, Biostatistics for Public health, Biostatistical Programming, Applied Math in Public Health/Biostatistics |
| 60 | Personal Health and Wellness | Personal Wellness, Personal and Community Health, Fitness, Physical Activity, Consumer Health, Healthy Living, Personal Health, Stress Management |
| 54 | Capstone Experience | Signature Experience: Capstone, Applied Capstone Seminar, Public Health Sciences Capstone, Practice of Evidence-Based Health: Capstone, Capstone Internship, Culminating Experience, Advanced Public Health Practice: Capstone |
| 54 | Occupational Health and Safety | Occupational Health, Occupational and Environmental Safety, Injury Prevention and Control, Health and Safety, Environmental Exposure Assessment, Risk Management, First Aid, Hazardous Materials health and Safety, Case Studies of Workplace Safety and Health, Health Promotion in the Workplace |
| 53 | Emergency Preparedness and Disaster Management | Emergency Response, Disaster Management, Risk Assessment, Responding to Emergencies, Crisis Management, States of Emergency, Foundations in Public Health Preparedness and Disaster Response, Health Emergencies in Large Populations |
| 49 | Chronic Disease and Infectious Disease | Disease Prevention, Infectious and Non-Communicable Disease, Disease and Injury, Disease Investigation, Ecology of Infectious Disease, Disease Management, Health Promotion and Disease Prevention, History of Disease, Immunology and Immunopathogenesis, Social History of Infectious Disease, HIV/AIDS, COVID-19, Emergent Threats, Bacteriological, Cancer |
| 48 | Substance Use, Addiction, and Violence Prevention | Drug Abuse and Prevention Education, Drug and Alcohol Issues, The Nature of Addiction, The opioid Epidemic, Tobacco as a Public Health Issue, Drug Use in American Society, Ethics in Addiction Services, Violence and Addiction, Violence and Public Health |
| 44 | Fieldwork/Practicum | Public Health Fieldwork, Field Experience in Health Science Public Health Field Placement, Fieldwork and Reflective Seminar, Field Practicum in Community Health, Peer Health Practicum, Practicum in College Sex Education, Practicum in Global Health |
| 41 | Community Nutrition | Child Nutrition, Nutrition for Disease Prevention, Nutrition and Epidemiology, Diet and Nutrition, Nutrition and Culture, Nutrition Concepts and Controversies, Nutrition in the Lifecycle, Nutrition and Sports Performance, Applied Nutrition |
| 37 | Biological Basis of Disease | Public Health Biology, Biological Foundations of Public Health, Biological Basis of Personal and Public Health, Human Diseases & Conditions, Introduction t o Human Health and Disease, Agent Host & Environment, Public Health Biology and Pathophysiology, Biology for Population Health |
| 36 | Health Communication | Public Health, Patient Communication, Healthcare Communication, Health Literacy, Communication for social and Behavioral Change, Effective Health Communication, Public Health Communication Skills, Communication in Public Health, Health and Wellness Communication |
| 35 | Public Health Economics and Finance | Economics and Decision making in Public Health, Public Health Finance and Management, Financial Administration of Public Health Projects, Fundraising and Finance in Public Health, Health Economics, |
| 34 | Food and Water Safety | Food Security, Sanitation, Food Access, Air Pollution, Water Resources, Agroecology, Food and Culture, Food Literacy, Urban Food Systems, Food Safety and Consumer Protection, Food Security in the US, Global Food Systems, Food and Culture, Geography of Health, Water and Air Quality, Water Treatment, Water Supply Safety |
| 34 | Sequential academic preparation courses | First Year Experience, The Sophomore Experience, Second-Year undergraduate Public Health Seminar, Public Health Senior Seminar, Senior Seminar - CHES Review, Thesis Research |
| 33 | Special Topics and Populations | Indigenous Health Issues, Emergency Public Health Topics, Special Topics in Public Health, International Relations in Public Health, Disabilities and Public Health, Rural Health, Native American health issues, Latino Health |
| 33 | Sexual Health and Reproductive Issues | Family Life and Sexuality, Reproductive Health and Medicine, Principles of Sexual Health Education, Sexual health Promotion, Society and Sex, Health, Sexuality and Family Life, Reproductive Health & Global Health |
| 28 | Population Health Intervention and Evaluation* | Program Evaluation, Quality Improvement, Needs Assessment, Implementing and Evaluation Health Programs, Assessment and Evaluation in Public Health Programs, Advanced Evaluation Methods, Health Program Evaluation, Health Program Assessment |
| 28 | Health Sciences | Foundations of Health Science, Health Science Terminology, Medical Terminology, Holistic Health, Alternative Health, Integrative Health, Health Genetics, Epigenetics, Health Privacy, Human Values and Science, Medical Science, Radiological Health, Nuclear Health |
| 23 | Law/Ethics in Healthcare | Medical Ethics, Health care ethics, Public Health Ethics, Ethics Policy & Law, Ethics Law & Politics, Ethics Law & Research, Bioethics for Healthcare |
| 20 | Mental Health | Mental and Behavioral Health Issues, Community Mental Health, Introduction to Public Mental Health, Population Mental Health and Well-being, Mental Health Issues in Public Health, Mental Health Services, Understanding Mental Health as Public Health |
| 19 | Technical Writing for Public Health | Technical Writing, Grant Writing, Proposal Writing, Prospectus, Proposal and Dissertation Writing, Scientific Writing |
| 16 | Milestones in the History of Public Health | Introduction to the History of Public Health, History of Public Health, From Cholera to Cancer: History and Challenges in Public Health, Historical Perspectives on Community Health, Critical History of Public Health in the US, Epidemics: From Plague to COVID |
| 16 | Community Health in Schools and Colleges | Contemporary Issues in College Health, College Health Promotion, Critical School Health Issues, Elementary School Health, Introduction to School Health, Strategies in School Health, School Health Educational Planning, Health in the School Environment |
| 14 | Media and Marketing | Mass media, social marketing, technology for social marketing, public health and popular culture, public health through film, public health media and technology, public health marketing |

**Appendix B. Undergraduate CEPH ASPPH course mapping**

**Appendix B1. Background Domains**

| **ASPPH** | **CEPH** | **Our course themes** |
| --- | --- | --- |
| **I. Background Domains** | **D10. Public Health bachelor’s degree Foundational Competencies** | These categories of CEPH requirements and ASPPH recommendations were not analysed due to the data collections methods used in the current study. |
| **A. Content area** | 2. Locate, use, evaluate, and synthesize public health information |  |
| Science |  |  |
| Social and Behavioral Science |  |  |
| Math/Quant |  |  |
| Humanities/Fine Arts |  |  |
| **B. Skill Areas** | 1. Communicate public health information, in both oral and written forms, through a variety of media and to diverse audiences |  |
| Communications |  |  |
| Information Literacy |  |  |

**Appendix B2. Public Health Domains**

| **ASPPH** | **CEPH** | **Our course themes (*n* = 2,259)** |
| --- | --- | --- |
| **II. Public Health Domains** | **D9. Public Health bachelor’s degree Foundational Domains** |  |
| 1. Overview of Public Health | 1. concepts and applications of basic statistics  3. the history and philosophy of public health as well as its core values, concepts, and functions across the globe and in society | - Foundations of Public Health (148) - History of Public Health (16) - Biostatistics (60) - Epidemiology (135) - Sequential academic preparation courses (34) - Global Health Issues (116) |
| 2. Roles and Importance of data in Public Health | 4. the basic concepts, methods, and tools of public health data collection, use, and analysis and why evidence-based approaches are an essential part of public health practice | - Health Data Analytics and Informatics (68) - Public Health Research Methods (83) |
| 3. Identifying and Addressing Population Health Challenges | 5. the concepts of population health, and the basic processes, approaches and interventions that identify and address the major health-related needs and concerns of populations | - Health Behaviour Theory and Practice (80) - Food and Water Access and Safety (34) |
| 4. Human Health | 2. the foundations of biological and life sciences  6. the underlying science of human health and disease, including opportunities for promoting and protecting health across the life course | - Chronic Disease and Infectious Disease (49) - Personal Health and Wellness (60) - Health, Gender, and the Human Lifespan (79) - Health Sciences (28) - Community Nutrition (41) - Biological Basis of Disease (37) |
| 5. Determinants of Health | 7. the socioeconomic, behavioral, biological, environmental, and other factors that impact human health and contribute to health disparities | - Social Determinants and Health Disparities (113) - Climate and Environmental Health (116) - Substance Use, Addiction, and Violence Prevention (48) - Sexual Health and Reproductive Issues (33) - Mental Health issues (20) |
| 6. Project Implementation | 8. the fundamental concepts and features of project implementation, including planning, assessment, and evaluation | - Population Health Program Design & Implementation (91) - Population Health Intervention and Evaluation (28) - Community Health in Schools and Colleges (16) |
| 7. Overview of the Health System | 9. the fundamental characteristics and organizational structures of the U.S. health system as well as the differences between systems in other countries | - US Health Systems and Policy Issues (77) |
| 8. Health Policy, Law, Ethics, and Economics | 10. basic concepts of legal, ethical, economic, and regulatory dimensions of health care and public health policy and the roles, influences, and responsibilities of the different agencies and branches of government | - Public Health Management, Policy, and Leadership (120) - Law/Ethics in Healthcare (23) - Public Health Economics and Finance (35) - Occupational Health and Safety (54) - Emergency Preparedness and Disaster Management (53) |
| 9. Health Communication | 11. basic concepts of public health-specific communication, including technical and professional writing and the use of mass media and electronic technology | - Health Education, Promotion, and Advocacy (89) - Technical Writing for Public Health (19) - Media and Marketing (14) - Health Communication (36) |

**Appendix B3. Cumulative Experience and Field Exposure**

| **ASPPH** | **CEPH** | **Our course themes (*n* = 2,259)** |
| --- | --- | --- |
| **III. Cumulative Experience and Field Exposure** | **D11. Public Health bachelor’s degree Cumulative & Experiential Activities** |  |
| 1. Cumulative Experience |  | - Capstone Experience (54) - Special Topics and Populations (33) |
| 2. Field Experience |  | - Independent Study, Internships, and Study Abroad (75) - Fieldwork/Practicum (44) |

**Appendix B4. Cross Cutting Areas**

| **ASPPH** | **CEPH** | **Our course themes** |
| --- | --- | --- |
| **IV. Cross Cutting Areas** | **D12. Public Health bachelor’s degree Cross-Cutting Concepts & Experiences** |  |
| 1. Advocacy for protection and promotion of the public’s health at all levels of society | 1. advocacy for protection and promotion of the public’s health at all levels of society | - Climate and Environmental Health - Health, Gender, and the Human Lifespan - Personal Health and Wellness - Community Nutrition - Special Topics and Populations |
| 1. Community dynamics | 2. community dynamics | - Health Education, Promotion, and Advocacy - Occupational Health and Safety - Population Health Intervention and Evaluation |
| 1. Critical thinking and creativity | 3. critical thinking and creativity | - Health Behavior Theory and Practice - Sequential academic preparation courses |
| 1. Cultural contexts in which public health professionals work | 4. cultural contexts in which public health professionals work | - Global Health Issues - Social Determinants and Health Disparities - Substance Use, Addiction, and Violence Prevention - Community Health in Schools and Colleges - Mental Health |
| 1. Ethical decision making as related to the self and society | 5. ethical decision making as related to self and society | - Food and Water Access and Safety - Sexual Health and Reproductive Issues - Law/Ethics in Healthcare |
| 1. Independent work and a personal work ethic | 6. independent work and a personal work ethic | - Independent Study, Internships, and Study Abroad - Capstone Experience - Technical Writing for Public Health |
| 1. Networking | 7. networking | - Fieldwork/Practicum - Health Communication - Media and Marketing |
| 1. Organizational dynamics | 8. organizational dynamics | - Population Health Program Design & Implementation - US Health Systems and Policy Issues - History of Public Health |
| 1. Professionalism | 9. professionalism | - Public Health Economics and Finance |
| 10. Research methods | 10. research methods | - Epidemiology - Public Health Research Methods - Health Data Analytics and Informatics - Biostatistics |
| 11. Systems thinking | 11. systems thinking | - Foundations of Public Health - Chronic Disease and Infectious Disease - Biological Basis of Disease - Health Sciences |
| 12. Teamwork and leadership | 12. teamwork and leadership | - Public Health Management, Policy, and Leadership - Emergency Preparedness and Disaster Management |
